# Fungi and Mycotoxins from Pre- and Poststorage Brewer's Grain Intended for Bovine Intensive Rearing

**DOI:** 10.5402/2012/396590

**Published:** 2012-10-15

**Authors:** L. A. M. Keller, C. M. Pereyra, L. R. Cavaglieri, A. M. Dalcero, C. A. R. Rosa

**Affiliations:** ^1^Departamento de Microbiologia e Imunologia Veterinária, Universidade Federal Rural do Rio de Janeiro, Rodovia BR 465 Km 7, 23890-000 Seropédica, RJ, Brazil; ^2^Conselho Nacional de Desenvolvimento Científico e Tecnológico (CNPq), Brasilia, DF, Brazil; ^3^Departamento de Microbiología e Inmunología, Universidad Nacional de Río Cuarto, Ruta 36 Km 601, Córdoba, 5800 Río Cuarto, Argentina; ^4^Consejo Nacional de Investigaciones Científicas y Técnicas (CONICET), Buenos Aires, Argentina

## Abstract

The aim of the study was to determine the mycobiota and natural levels of mycotoxins such as aflatoxin B_1_ (AFB_1_), ochratoxin A (OTA), fumonisin B_1_ (FB_1_), and deoxynivalenol (DON) present in brewers grains pre- and poststored intended for bovine intensive rearing. Poststored (80%) samples had counts higher than 1 × 10^4^ colony-forming units (CFU/g). *Cladosporium* spp. and *Aspergillus* spp. were isolated at high frequencies. *Aspergillus flavus* was the prevalent isolated species. Prestored (70%) and poststored (100%) samples showed AFB_1_ levels over the recommended limits (20 **μ**g/Kg), and OTA levels were below the recommended limits (50 **μ**g/Kg) while pre- and poststored samples did not show FB_1_ and DON natural contamination levels. The presence of mycotoxins in this substrate indicates the existence of contamination. Regular monitoring of feeds is required in order to prevent chronic and acute toxic syndromes related to this kind of contamination.

## 1. Introduction 

The use of agroindustrial residues as a food supplement for animal production plays a significant economic role due to the availability and versatility of these materials. Brewer's grains (beer industry residue) are an interesting alternative option as feeding for animal production, being a rich source of protein and fibber at a low price [1, 2]. Inadequate management of raw materials during storage can result in excessive moisture or dryness, condensation, heating, leakage of rainwater, and insect infestation, leading to undesirable growth of fungi [3]. Worldwide, the contamination of animal feed and the potential contamination of animal meat by mycotoxins represent a serious hazard to humans and animals. Mycotoxins are toxic, chemically diverse secondary substances or metabolites produced by a wide range of fungi. They are mainly produced by *Aspergillus*, *Penicillium,* and *Fusarium* genera [4]. Due to the diversity of their toxic effects and their synergetic properties, mycotoxins are considered as risky to the consumers of contaminated foods and feeds [5]. Aflatoxins (AFs), the fungal metabolites produced by some strains of *A. flavus *and *A. parasiticus, *are of great concern because of their detrimental effects on the health of humans and animals, including carcinogenic, mutagenic, teratogenic, and immunosuppressive effects [6, 7]. Ochratoxin A (OTA) is one of the most common and dangerous mycotoxin in food and feed, naturally produced by *A. ochraceus*, *A. carbonarius* and *A. niger* aggregate mainly in tropical regions, and *P. verrucosum* in temperate areas [8–10]. This toxin has a potent toxicity, and the nephrotoxic, hepatotoxic, teratogenic, carcinogenic, and immunosuppressive effects have been demonstrated in all mammalian species [11]. Fumonisins (FBs), produced by *Fusarium verticillioides *and *F. proliferatum,* occur worldwide and are predominantly found in maize and in maize-based animal feeds. Fumonisin B_1_ (FB_1_) is the most common and the most thoroughly studied, causing toxicities in animals such as equine leukoencephalomalacia (ELEM) and porcine pulmonary edema (PPE), diseases long associated with the consumption of mouldy feed by horses and pigs, respectively [12]. Deoxynivalenol (DON) or vomitoxin is a commonly occurring mycotoxin produced primarily by *F. graminearum* and *F. culmorum* [13]. This toxin can cause vomiting, feed refusal, gastrointestinal irritation, and immunosuppression [14].

Previous studies performed in Brazil determined the fungal biota as well as the presence of different mycotoxins in brewer's grain and barley rootlets intended for cattle and pigs [15–18]. There are no data about the contamination with fungi and mycotoxins in brewer's residue stored in farms in a similar manner of trench type silos during 3 months. Therefore, the aims of this work were to determine mycobiota occurrence and to evaluate AFs, OTA, FBs, and DON incidence on pre- and poststorage brewer's grains. 

## 2. Materials and Methods

### 2.1. Characteristic of Storage

Brewer's grains were transported from brewery industry to farms in trucks, deposited in five (5) structures similar to a trench type and stored in large pits dug into ground (1 m) and covered with a black plastic sheet. Storage and compaction was performed during 3 months, and brewer's grains were kept closed until to be used. The removal of material for animal feeding was made by shovelling.

### 2.2. Samples Source

Brewer's grain samples were collected from bovine intensive rearing (feedlot) 2 farms in São Paulo State, Brazil. These samples were collected at different times: day zero (0) (immediately deposited) and after 90 days of storage (before feeding animals). To guarantee a correct sampling, each structure similar to trench type was imaginary divided along its length into three equal parts with four sections each: upper, lower, border, and middle sections. Six subsamples (500 g) were collected from each section to obtain a total of three kilograms sample. A total of 100 samples (3 kg each) of brewer's grains were taken at different times: 50 were taken at day 0, and 50 samples were taken at day 90. Samples were properly packed in bags and immediately sent to the laboratory. Samples were immediately processed for physical and mycological analyses and kept at −4°C until mycotoxins analyses.

### 2.3. Physical Properties of Samples

The pH and dry matter percentage for 100 g of each sample were determined according to Ohyama et al. [19]. 

### 2.4. Mycological Analysis

The quantitative enumeration of fungi as colony-forming units per gram of food (CFU/g) was performed using the surface-spread method described by Pitt and Hocking [20]. Ten grams of each sample were homogenized in 90 mL distilled water solution for 30 min in an orbital shaker. Serial dilutions (from 10^−2^ to 10^−5^) were made, and 0.1 mL aliquots were inoculated in duplicates onto the media dichloran that rose bengal chloranphenicol agar (DRBC) for estimating total culturable fungi [21] and dichloran 18% glycerol agar (DG18) that favors xerophilic fungi development. The plates were incubated at 25°C for 5–7 days. All samples were also inoculated onto Nash and Snyder agar (NSA) to enumerate *Fusarium* species [22]. Nash-Snyder plates were incubated at 24°C for 7 days under a 12 h cold white/12 h black fluorescent light photoperiod. Only plates containing 10–100 CFU were used for counting, with results expressed as CFU per gram of sample. On the last day of incubation, individual CFU/g counts for each colony type considered to be different were recorded. Colonies representative of *Aspergillus* and *Penicillium *were transferred for subculturing to tubes containing malt extract agar (MEA) whereas *Fusarium* spp. were transferred for subculturing to plates containing carnation leaf agar (CLA). Fungal species were identified according to Klich [23], Nelson et al. [22], and Samson et al. [24]. The results were expressed as isolation frequency (% of samples in which each genus was present) and relative density (% of isolation of each species among the same genera).

### 2.5. Mycotoxins Detection and Quantification

#### 2.5.1. Aflatoxin B_1_ and OTA Determination

The extraction of AFB_1_ was determined according to Soares and Rodriguez-Amaya [25]. Quantitative evaluation was made using high-performance liquid chromatography (HPLC). The detection limit of the technique for AFB_1_ was 1.0 *μ*g/kg. 

#### 2.5.2. Fumonisin B_1_ Determination

A commercially available enzyme-linked immunosorbent assay (ELISA) plate kit (Beacon Analytical Systems Inc., Portland, USA) was applied for the extraction and quantification of FB_1_. Mycotoxin extraction and testing were performed according to manufacturer's introductions. A 20 g portion of each sample was extracted with 100 mL methanol:water (70 : 30, v/v) during 3 min into a blend jar. The mixture was filtered through filter paper Whatman N° 4 (Whatman, Inc., Clifton, NY, USA) and an aliquot taken and placed into a culture plate. Detection limit of the technique was 0.3 *μ*g/g.

#### 2.5.3. Deoxynivalenol Determination

An ELISA tube kit (Beacon Analytical Systems Inc., Portland, USA) was also applied for the extraction and quantification of DON. Mycotoxin extraction and testing (ELISA) were performed according to manufacturer's introductions. A 20 g portion of each sample was extracted with 100 mL distilled water during 3 min into a blend jar. The mixture was filtered through filter paper Whatman N° 4 (Whatman, Inc., Clifton, NY, USA) and an aliquot taken and placed into a culture tube. Detection limit of the technique was 0.5 *μ*g/g.

#### 2.5.4. Statistical Analyses

Statistical analysis of data was by the general linear models model (MLGM). Fungal counts were transformed to log_10_ (*x* + 1). Means obtained from CFU/g mycotoxin analyses were compared using Fisher's protected LSD test.

## 3. Results

### 3.1. Chemical and Physical Properties of Samples

Table 1 shows the physical properties of the sorghum samples. The pH mean levels ranged from 5.7 to 6.0 in prestored brewer's grain while the values of pH, from poststored were from 4.5 to 5. In both types of samples, dry matter values were from 39.7 to 41%. 

### 3.2. Mycological Survey

Table 2 shows fungal counts from pre- and poststored brewer's grains in different culture media. Total fungal count analyses from prestored shown values with means ranging from 1.7 × 10^3^ to 2.9 × 10^3^ CFU/g and 1.5 × 10^3^ to 1.8 × 10^3^ CFU/g in DRBC and DG18, respectively. Eighty percent of poststored samples had counts higher than 1 × 10^4^ CFU/g. Means varied from 2.5 × 10^4^ to 2.3 × 10^5^ CFU/g in DRBC and from 6.2 × 10^3^ to 1.5 × 10^5^ CFU/g in DG18. There were significant differences between pre- and poststored brewer's grain samples. No statistically significant differences were found between different layer of the silo prestored brewer's grain in DRBC and DG18 while there were significant differences between fungal counts from poststored samples (*P* < 0.05).

**Figure 1:**
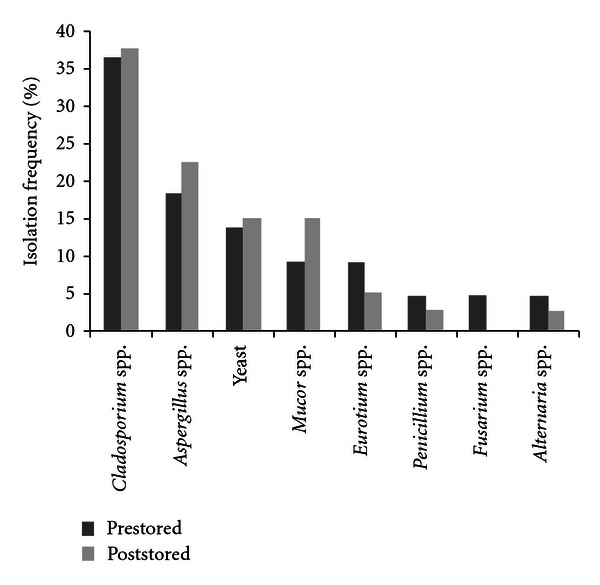
Isolation frequency of fungal genera (%) from pre- and poststored brewer's grains samples.

Figure 1 shows the isolation frequency of fungal genera (%) from pre- and poststored brewer's grain samples.* Cladosporium* spp., *Aspergillus* spp., *Mucor* spp., and yeasts were isolated at high frequencies. *Eurotium* spp., *Penicillium* spp. and *Alternaria* spp. were isolated at low frequencies. *Fusarium* spp. were isolated only from poststored brewer's grain samples. 

**Figure 2:**
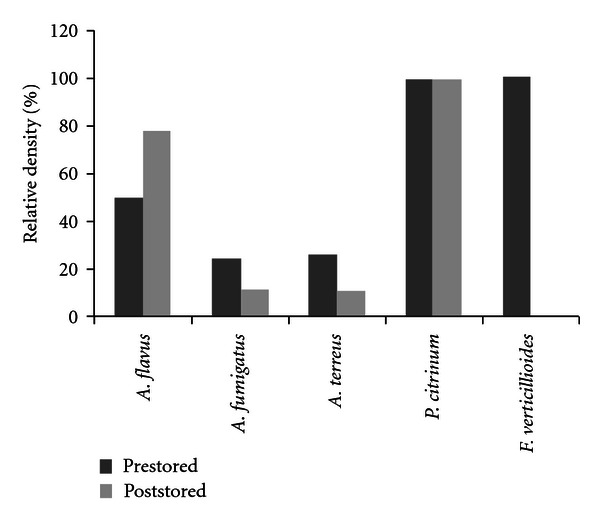
Relative density (%) of *Aspergillus* spp., *Penicillium* spp. and *Fusarium* spp. isolated from pre- and poststored brewers grains samples.

Figure 2 shows the relative density of isolated* Aspergillus* spp., *Penicillium* spp., and *Fusarium* spp. from pre- and poststored brewer's grain samples. Three *Aspergillus* spp. were isolated. *Aspergillus flavus* was the prevalent isolated species, followed by *A. fumigatus* and *A. terreus*. *Aspergillus flavus* was isolated at levels that ranged from 50 to 78% for pre- and poststored samples, respectively. While *A. fumigatus* and *A. terreus* were isolated from pre- and poststored samples. *Penicillium citrinum* was the only species isolated within this genus. *Fusarium verticillioides* was only present in prestored brewer's grain samples. 

### 3.3. Determination of Mycotoxins

Table 3 shows the AFB_1_ and OTA levels found in pre- and poststored brewer's grain samples. Pre- and poststored samples did not show FB_1_ and DON natural contamination levels. Four percent of pre- and poststored samples were contaminated with AFB_1_ at levels that varied from 10 to 35 *μ*g/Kg and 24 to 47 *μ*g/Kg, respectively. Seventy percent of prestored and all poststored samples (100%) showed AFB_1_ levels over the recommended limits (20 *μ*g/Kg). None of the analyzed of prestored samples showed OTA levels. While 5% of poststored samples were contaminated with average levels of 9.8 *μ*g/Kg. However, none of these samples were contaminated with OTA levels over the recommended limits (50 *μ*g/Kg). No statistically significant differences were found between pre- and poststored brewer's grain for AFB_1_ and OTA contamination (*P* < 0.05).

## 4. Discussion 

Mycobiota and natural occurrence of AFB_1_, OTA, FB_1_, and DON in pre- and poststored brewer's grain were studied.

Physical properties of brewer's grain samples showed that there was no difference in dry matter comparing pre- and poststored brewer's grains. The dry matter content is one of the main factor for well-preserved samples. The ideal values of this parameter are between 26 and 38% with pH around 4.0 [27]. The physical factor that assures the preservation is pH. The pH difference between pre- and poststored samples is due to the acidification of carbohydrates present in the raw material by microorganisms present in this ecosystem. In this work, this substrate was acidified through time, and the pH values in poststored brewers' grain were between 4.5 to 5.0 after 90 days of storage. 

In this study, the average of fungal colony counts from all prestored brewer's grain samples had counts lower than the maximum proposed limit (1 × 10^4^ CFU/g) [26]. However, poststored brewer's grain samples had high values, which were over the maximum of the recommended limits. These results suggest a high fungal activity that could affect the palatability of feed and reduce the animal nutrients absorption, determining a low-quality substrate [28, 29]. Simas et al. [15] and Rosa et al. [17] studied the same substrate intended for dairy cattle feed. They found media counts of 1 × 10^3^ CFU/g and 6 × 10^5^ CFU/g in potato dextrose agar and DRBC media, respectively. Cavaglieri et al. [18] obtained counts ranging between 1 × 10^3^ and 1 × 10^6^ CFU/g in DRBC; however, they studied other waste derived from processing of barley intended for pigs (barley rootlet). This substrate was storage between 8 and 15 days while in this study the period was 90 days. 

In this work, *Cladosporium* spp. and *Aspergillus* spp. were the most prevalent genera isolated from pre- and poststored samples. Similar percentages of *Aspergillus* spp. were found by Cavaglieri et al. [18] in barley rootlets; in addition they found *Fusarium* spp. as the prevalent genus. In this study, the scarce presence of *Fusarium* sp. may be the result of brewer's grain storage and processing conditions. These conditions may have favoured the development of storage and contaminant fungi instead of those known as field fungi, which include the genus *Fusarium*, more frequently found on recently harvested grain than on processed and stored ones [30]. Several studies have proved that *Aspergillus* and *Penicillium* genera were predominant in brewer's such as Simas et al. [15], Rosa et al. [17], and Gerbaldo et al. [31]. A high frequency of yeasts was also found. The significance of yeasts, which were frequently isolated, is not known in this substrate.

In this study, *A. flavus* was the most prevalent species followed by *A. fumigatus* and *A. terreus*. These results agree with those of Gerbaldo et al. [31] who reported high percentages of *A. flavus* and *A. fumigatus* in brewer's grains intended for pigs in Argentina. Rosa et al. [17] found *A. niger* aggregate as prevalent followed by *A. ochraceus, A. terreus *and *A. flavus* from dairy cattle feed. *Penicillium citrinum* was only species of *Penicillium* genus isolated. Previous studies in the some substrate have demonstrated high frequency of *P. citrinum* together with *P. funiculosum*, *P. janthinellum*, *P. rugulosum,* and *P. viridicatum* [17]. *Fusarium verticillioides* was isolated at low frequency in our study. Cavaglieri et al. [18] studied barley rootlets as feed for pigs. They found *F. verticillioides* as the only species within *Fusarium* genus, but at high frequency. Other researchers did not identify species of *Fusarium* sp. from the same substrate of this work [15, 17, 31]. 

Scientific reports on the contamination of brewer's grain with mycotoxins in Brazil are scarce. Simas et al. [15] studied the presence of AFB_1_ and OTA in this substrate. In this study, levels of AFB_1_ found from prestored samples were higher than those obtained by Simas et al. [15]. Considering thevast territoryof Brazil,this may be dueto differentclimaticconditions betweenthe two states.

Regulations on standard products in the animal feed sector established that the current maximum permitted level for AFB_1_ is 20 *μ*g/Kg [26]. In this work, 75% and 100% of the samples contaminated at 0 and 90 days of storage, respectively, showed AFB_1_ levels higher than the recommended limits for feedstuffs. The OTA concentrations were observed in samples derived from poststored samples. Rosa et al. [17] found higher amounts of OTA in samples of brewer's grains. In this work, OTA levels were below the recommended limit which is 50 *μ*g/Kg [26]. The presence of this mycotoxin in this substrate indicates the existence of contamination, a fact that would require periodic monitoring. Brewer's grains samples did not show FB_1_ and DON contamination. Our results did not agree with Batatinha et al. [16] and Cavaglieri et al. [18] who found FB_1_ in brewer's grains and barley rootlets, at levels that ranged from 198 to 295 *μ*g/Kg and from 564 to 1383 *μ*g/Kg, respectively. Preharvest contamination of the barley crop could be considered possible, barley could support *F. verticillioides*/*F. proliferatum* growth when grain is remoistened during the germination and malting process, and it might even continue during storage prior to use, providing that the water activity remained high. The malting process requires water to allow barley germination. If fumonisins were present, they could be diluted during the steeping process. No information is available about the study of DON in this substrate. While this report does not detect this toxin, this is the first study to investigate its presence. 

The presence of mycotoxins in these substrates indicates the existence of contamination. Inadequate storage conditions promote the proliferation of mycotoxin-producing fungal species. Regular monitoring of feeds is required in order to prevent chronic and acute toxic syndromes related to this kind of contamination. 

## Figures and Tables

**Table 1:** Physical properties from pre- and poststored brewer's grains in several section of silo.

	pH		Dry matter (%)	
Section of silo	Means ± SD		Means ± SD	
	Pre	Post	Pre	Post
Upper	5.7 ± 1.0	4.6 ± 0.8	46 ± 0.07	40 ± 0.13
Middle	6.0 ± 0.3	4.5 ± 0.4	37 ± 0.11	41 ± 0.13
Lower	5.9 ± 0.4	5.0 ± 0.8	44 ± 0.09	41 ± 0.12
Border	6.0 ± 0.35	4.6 ± 0.7	37 ± 0.12	37 ± 0.11

SD: standard deviation.

**Table 2:** Fungal counts (CFU/g) from pre- and poststored brewer's grains samples in DRBC and DG18 culture media.

		Fungal counts (CFU/g)		
Samples	Section of silo	Mean-Range*		Contaminated samples (%) over GMP (2008) [26] limits
		DRBC	DG18	
	Upper	2.9 × 10^3a^	1.6 × 10^3a^	
		(3.3 × 10^2^–6.1 × 10^3^)	(1 × 10^2^–4.8 × 10^3^)	
	Lower	2.2 × 10^3a^	1.8 × 10^3a^	
Prestored		(2.8 × 10^2^–5.2 × 10^3^)	(3.1 × 10^2^–4.4 × 10^3^)	0 %
	Border	2.5 × 10^3a^	1.5 × 10^3a^	
		(2.8 × 10^2^–6.1 × 10^3^)	(1 × 10^2^–4.8 × 10^3^)	
	Middle	1.7 × 10^3a^	1.8 × 10^3a^	
		(1 × 10^3^–2.8 × 10^3^)	(3.1 × 10^2^–4.4 × 10^3^)	

	Upper	1.3 × 10^5abc^	1.5 × 10^5abc^	
		(3.1 × 10^3^–3 × 10^5^)	(1.6 × 10^3^–4.3 × 10^5^)	
	Lower	2.5 × 10^4abc^	2.3 × 10^4ab^	
Poststored		(1.2 × 10^3^–5 × 10^4^)	(3.4 × 10^3^–6.7 × 10^4^)	80%
	Border	1.8 × 10^5bc^	6.2 × 10^3a^	
		(1 × 10^3^–1.4 × 10^6^)	(1.2 × 10^3^–2.1 × 10^4^)	
	Middle	2.3 × 10^5c^	5.2 × 10^4abc^	
		(1.3 × 10^4^–5.2 × 10^5^)	(1.3 × 10^4^–1.5 × 10^5^)	

^∗^Mean values of counts. Minor and major values count. Detection limit: 1 × 10^2^ CFU/g. Maximum recommended level: 1 × 10^4^ CFU/g [26]. DRBC: dichloran rose bengal chloranphenicol. DG18: dichloran glycerol 18%. Letters in common are not significantly different according to Fisher's protected LSD test (*P* < 0.05).

**Table 3:** Incidence of aflatoxin B_1_ and ochratoxin A in pre- and poststored brewers grains samples.

			Mycotoxins			
Samples		AFB_1_ (*μ*g kg^−1^)			OTA (*μ*g kg^−1^)	
	Mean levels	Range	(%)*	Mean levels	Range	(%)*
Pre^1^	25.8^a^	10–35	8	ND^a^	—	—
Post^2^	38^a^	24–47	10	9.8^a^	2–25	5

^1^Prestored brewers grains; ^2^Poststored brewers grains; *Percentage of samples contaminated with mycotoxin (%). ND: not detected. Values with letters in common are not statistically significant, according to test of LSD (*P* ≤ 0.05).

## References

[1] Lopez-Diaz TM, Flannigan B (1997). Production of patulin and cytochalasin E by *Aspergillus clavatus* during malting of barley and wheat. *International Journal of Food Microbiology*.

[2] Fagundes MH Sementes de cevada. http://www.conab.gov.br/OlalaCMS/uploads/arquivos/ee9b65650e13403f19f724263401b977..pdf.

[3] Dos Santos VM, Dorner JW, Carreira F (2003). Isolation and toxigenicity of *Aspergillus fumigatus* from moldy silage. *Mycopathologia*.

[4] Akande KE, Abubakar MM, Adegbola TA, Bogoro SE (2006). Nutritional and health implications of mycotoxins in animal feeds. *Pakistan Journal of Nutrition*.

[5] Yiannikouris A, Jouany JP (2002). Mycotoxins in feeds and their fate in animals: a review. *Animal Research*.

[6] Khanafari A, Soudi H, Miraboulfathi M (2007). Biocontrol of *Aspergillus flavus* and aflatoxin B_1_ production in corn. *Iranian Journal of Environmental Health Science and Engineering*.

[7] Murthy GS, Townsend DE, Meerdink GL, Bargren GL, Tumbleson ME, Singh V (2005). Effect of aflatoxin B_1_ on dry-grind ethanol process. *Cereal Chemistry*.

[8] Abarca ML, Accensi F, Bragulat MR, Cabañes FJ (2001). Current importance of ochratoxin A-producing *Aspergillus* spp. *Journal of Food Protection*.

[9] Magnoli C, Astoreca A, Ponsone L (2004). Survey of mycoflora and ochratoxin A in dried vine fruits from Argentina markets. *Letters in Applied Microbiology*.

[10] Magnoli C, Astoreca A, Ponsone L, Fernández-Juri MG, Chiacchiera S, Dalcero A (2006). Ochratoxin A and the occurrence of ochratoxin A-producing black aspergilli in stored peanut seeds from Córdoba, Argentina. *Journal of the Science of Food and Agriculture*.

[11] Karlovsky P (1999). Biological detoxification of fungal toxins and its use in plant breeding, feed and food protection. *Natural Toxins*.

[12] Voss KA, Smith GW, Haschek WM (2007). Fumonisins: toxicokinetics, mechanism of action and toxicity. *Animal Feed Science and Technology*.

[13] Rotter BA, Prelusky DB, Pestka JJ (1996). Toxicology of deoxynivalenol (vomitoxin). *Journal of Toxicology and Environmental Health*.

[14] Haschek WM, Voss KA, Beasley VR, Haschek WM, Roussex CG, Wallig MA (2002). Selected mycotoxins affecting animal and human health. *Handbook of Toxicologic Pathology*.

[15] Simas MMS, Botura MB, Correa B (2007). Determination of fungal microbiota and mycotoxins in brewers grain used in dairy cattle feeding in the State of Bahia, Brazil. *Food Control*.

[16] Batatinha MJM, Simas MMS, Botura MB, Bitencourt TC, Reis TA, Correa B (2007). Fumonisins in brewers grain (barley) used as dairy cattle feed in the State of Bahia, Brazil. *Food Control*.

[17] Rosa CAR, Cavaglieri LR, Ribeiro JMM (2008). Mycobiota and naturally-ochratoxin A in dairy cattle feed from Rio de Janeiro State. *World Mycotoxin Journal*.

[18] Cavaglieri LR, Keller KM, Pereyra CM (2009). Fungi and natural incidence of selected mycotoxins in barley rootlets. *Journal of Stored Products Research*.

[19] Ohyama Y, Masaki S, Hara S (1975). Factors influencing aerobic deterioration of silages and changes in chemical composition after opening silos. *Journal of the Science of Food and Agriculture*.

[20] Pitt JI, Hocking AD (1997). *Fungi and Food Spoilage*.

[21] Abarca ML, Bragulat MR, Castellá G, Cabañes FJ (1994). Ochratoxin A production by strains of *Aspergillus niger var. niger*. *Applied and Environmental Microbiology*.

[22] Nelson PE, Toussoun TA, Marasas WFO (1983). *Fusarium Species: An Illustrated Manual for Identification*.

[23] Klich MA (2002). *Identification of Common Aspergillus Species*.

[24] Samson RA, Van reenen-Hoekstra ES, Frisvad JC, Filtenborg O (2000). *Introduction to Food and Airborne Fungi*.

[25] Soares LMV, Rodriguez-Amaya DB (1989). Survey of aflatoxins, ochratoxin A, zearalenone, and sterigmatocystin in some Brazilian foods by using multi-toxin thin-layer chromatographic method. *Journal of the Association of Official Analytical Chemists*.

[27] Silva JM http://www.cnpgc.embrapa.br/publicacoes/divulga/GCD51.html.

[28] Ogundero VW (1987). Toxigenic fungi and the deterioration of nigerian poultry feeds. *Mycopathologia*.

[29] Martins HM, Martins ML (2001). Mycological quality evaluation of bovine feedstuffs (Portugal: 1996–1999). *Revista Portuguesa de Ciências Veterinárias*.

[30] Lillehoj EB (1973). Feed sources and conditions conducive to production of aflatoxin, ochratoxin, Fusarium toxins, and zearalenone. *Journal of the American Veterinary Medical Association*.

[31] Gerbaldo GA, Pereyra CM, Cavaglieri LR (2011). Surveillance of aflatoxin and microbiota related to brewers grain destined for swine feed in Argentina. *Veterinary Medicine International*.

